# Understanding sex differences in neuroimaging biomarkers and clinical trials

**DOI:** 10.1002/alz.090945

**Published:** 2025-01-09

**Authors:** Rachel F. Buckley

**Affiliations:** ^1^ Brigham and Women’s Hospital and Department of Neurology, Massachusetts General Hospital, Harvard Medical School, Boston, MA USA

## Abstract

The most recent Alzheimer’s clinical trials, including those which reported successful outcomes, use neuroimaging biomarkers of both amyloid and tau for screening participants and demonstrating a treatment effect on pathology. Some of these trials, notably Lecanemab, hint at a potential sex bias in treatment outcome, alluding to major implications for clinical practice when recommending treatment options. Sex differences in treatment response are not surprising given that women are at greater risk of progression to AD dementia, particularly if they carry *APOE*e4. Clinical trials, however, do not tend to sex disaggregate their topline results. Over the past 5 years, many independent studies across the world have reported sex differences in neuroimaging biomarkers of tau through positron emission tomography (PET). Women, primarily those with higher levels of amyloid, show significantly higher tau signal in the medial temporal and lateral temporal regions of the brain than men, after adjusting for age. This sex difference is evident not only in clinically normal middle and older‐aged adults, but also in diverse populations, and those with clinical impairment. Sex differences in tau‐PET also translate to faster rates of cognitive decline in women than men. Most recently, our group conducted a meta‐analysis of 1,286 unimpaired adults from four longitudinal cohorts including Alzheimer’s Disease Neuroimaging Initiative, Harvard Aging Brain Study, Wisconsin Registry of Alzheimer’s Prevention and Mayo Clinic Study of Aging. Among individuals with elevated baseline amyloid, women exhibited significantly faster tau accumulation in the medial and lateral temporal gyrus. This is some of the strongest evidence to date to suggest that not only could screening for clinical trials be effected by sex differences, but that treatment response – impacted by lowering pathological burden – could be highly sex‐specific. In this talk, we will present simulated data to demonstrate the issue of sex‐biases in tau baseline and accumulation levels to influence treatment response outcomes. We sit at a junction in the field where knowledge from observational studies of sex differences in AD neuroimaging biomarkers has not yet filtered through to clinical trials. This will be critical to ensure that both sexes are well served by treatment outcomes.

